# SARS‐CoV‐2 lgM/lgG antibody detection confirms the infection after three negative nucleic acid detection

**DOI:** 10.1111/jcmm.15275

**Published:** 2020-05-19

**Authors:** Hua Li, Jue Pan, Yi Su, Beili Wang, Junbo Ge

**Affiliations:** ^1^ Department of Cardiology Zhongshan Hospital Shanghai Institute of Cardiovascular Diseases Fudan University Shanghai China; ^2^ Department of Infectious Diseases Zhongshan Hospital Fudan University Shanghai China; ^3^ Department of Laboratory Medicine Zhongshan Hospital Fudan University Shanghai China

**Keywords:** SARS‐CoV‐2, lgM/lgG antibody detection, novel coronavirus

## Abstract

An ongoing outbreak of viral pneumonia was caused by a novel coronavirus in China in 2019. By March 19, over 200 thousand confirmed cases of SARS‐CoV‐2 infection and over 9000 deaths have been reported throughout the world. For this infectious disease, nucleic acid detection is still the gold standard for pathogenic detection. However, nucleic acid detection takes a long time and has relatively high "false negative"; therefore, we need urgently a convenient and accurate detection method to make up for this deficiency. In this article, we will show such technical characteristics of lgM/lgG serum antibody detection, compared with nucleic acid detection.

## 1 METHODS

### 1.1 RT‐PCR detection of SARS‐CoV‐2

The real‐time RT‐PCR kit was used to detect the nucleic acid of SARS‐CoV‐2. The primers and probes were designed for the sequences of both ORF1ab and N genes according to the COVID‐19^1^, ^2^, ^3^ Technical Guidelines for Laboratory Testing issued by National Health Commission of the People's Republic of China. For ORF1ab gene, the forward primer (F) was “CCCTGTGGGTTTTACACTTAA” and reverse primer (R) was “ACGATTGTGCATCAGCTGA”, the probe sequence was “5'‐FAM‐CCGTCTGCGGTATGTGGAAAGGTTATGG‐BHQ1‐3'”. For N gene, the forward primer was “GGGGAACTTCTCCTGCTAGAAT” and reverse primer was “CAGACATTTTGCTCTCAAGCTG”. The probe sequence was “5'‐FAM‐TTGCTGCTGCTTGACAGATT‐TAMRA‐3'”. Double positive for both ORF1ab gene and N gene was indicated to be positive.

### 1.2 Colloidal gold immunochromatography assay

The IgM antibody and IgG antibody against SARS‐CoV‐2 in serum samples were tested using colloidal gold immunochromatography assay kits (Bestnovo), according to the manufacture's instruction. Briefly, the IgM/IgG antibody in the human serum sample (10 μL) is first identified and then bound nitrocellulose membrane‐coated SARS‐CoV‐2 antigen, which is further combined with colloidal gold‐labelled mouse‐anti‐human IgM/IgG, triggering the colloidal gold colour development reaction.

## 2 RESULTS

Ten days before Jan 25, 2020, a‐46‐year‐old man, with a history of good physical health all along and not visiting Wuhan recently, contacted with one patient who had been confirmed SARS‐CoV‐2 infection on 24 January 2020. On 25 January 2020, he had developed fever, chill and myalgia. The next day he visited a local clinic in Shanghai with body temperature of 38.7°C. With the laboratory data (Table S1), he was primary diagnosed as a common cold with cefuroxime treatment and then went home. On January 28, he suddenly had high fever of 39°C and developed cough and expectoration. Thereupon, he immediately came to the fever clinic of one large comprehensive hospital.

**Figure 1:**
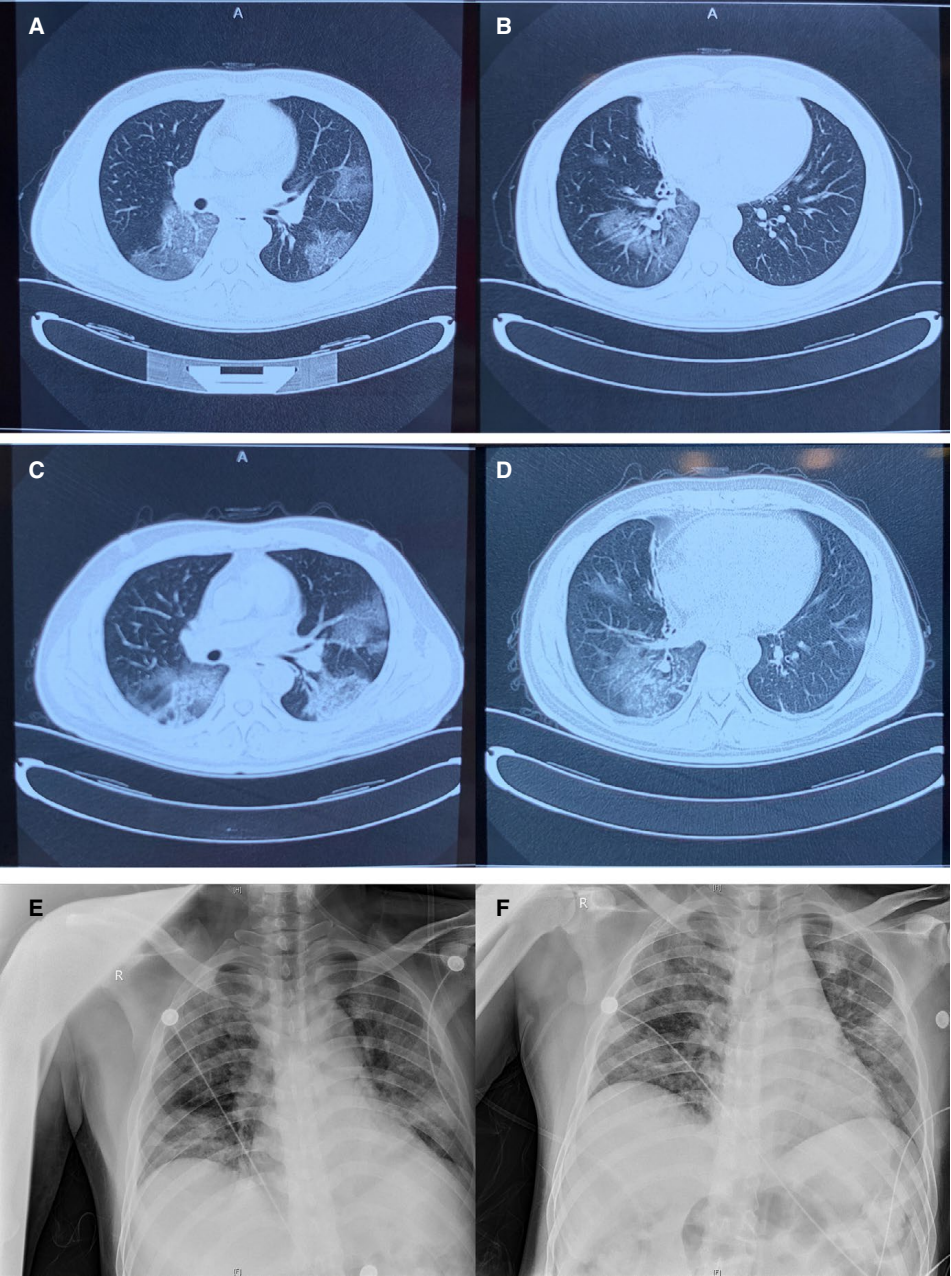
Chest imaging of the patient. A, B, HRCT scans taken on January 28, 2020 showed bilateral, multiple, ground‐glass opacities. C, D, HRCT scans taken on January 30, 2020 showed lung lesions progressed. E, F, The beside chest radiography taken on 3 February 2020 and 17 February 2020, separately

**Figure 2:**
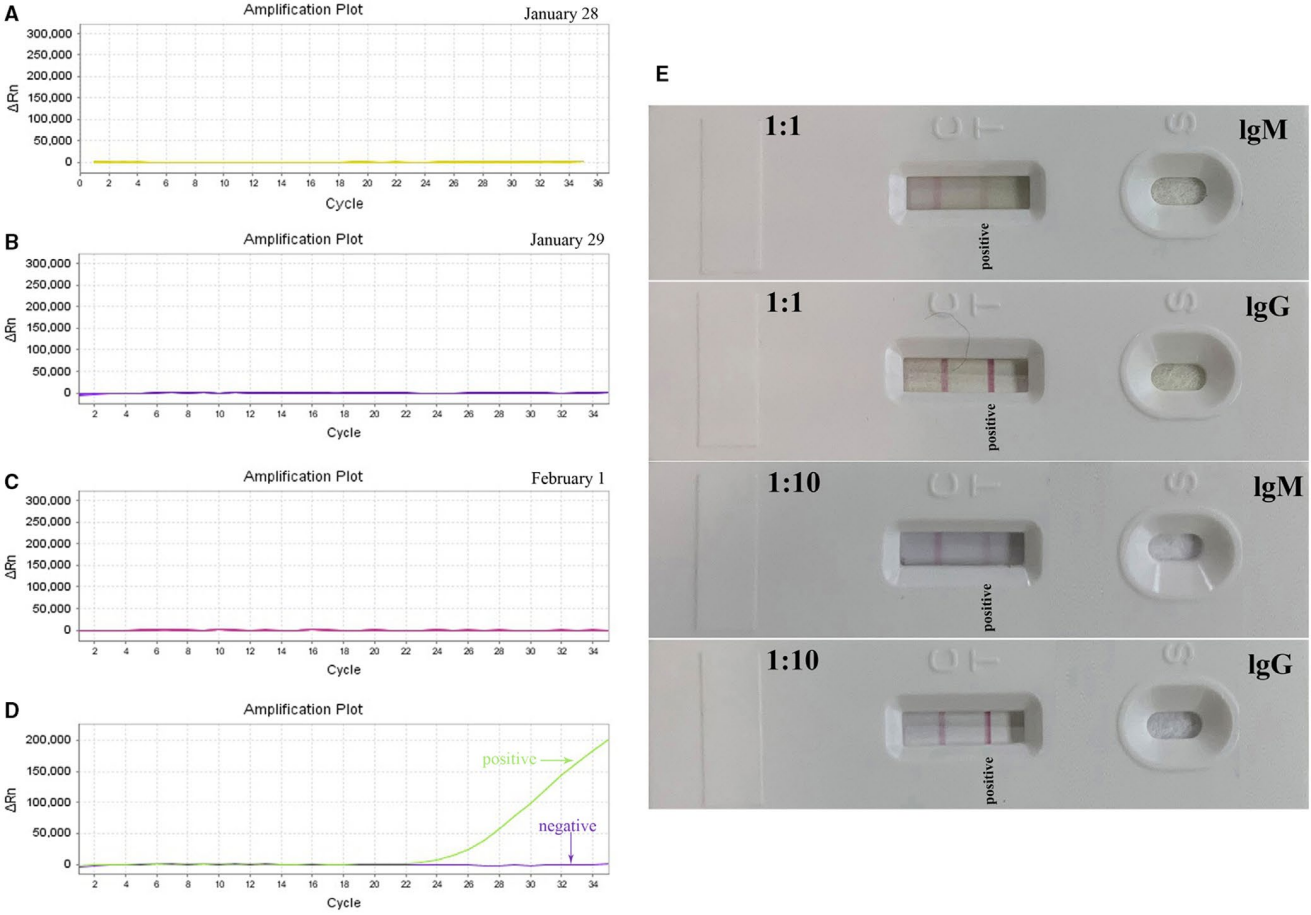
Biological detection of the patient. A‐C, Negative RT‐PCR results of the case on January 28, January 29 and February 1. D, The positive curve of RT‐PCR result of 2019‐nCoV infection. E, 2019‐nCoV specific IgM and lgG antibody was tested by colloidal gold strips on March 2, with both positive results

High‐resolution computed tomography (HRCT) on January 28 (day 3 of the disease) showed multiple, ground‐glass opacities located in both lungs (Figure 1A,B). Subsequently, throat swabs were obtained from the patient twice, however, both tested negative for SARS‐CoV‐2 by real‐time RT‐PCR assay (Figure 2A,B). Through synthetical consideration, he was isolated as a suspected patient with SARS‐CoV‐2 infection. On January 30, he complained of breathing difficulty on admission and oxygen saturation (SpO2) appeared decreased under the condition of nasal cannula (2 L/min). Emergent HRCT was taken with the result of lung lesions progressed (Figure 1C,D). To relieve his suffering, high flow oxygen supplementation via nasal cannula (15 L/min) was administered.

To make clear the pathogenic diagnosis for the patient, his blood, swab and faeces were all tested by real‐time RT‐PCR assay on February 1, but still negative (Figure 2C). On February 2, His oxygen saturation decreased obviously, and the situation had gone from bad to worse, with the admission of high flow nasal cannula 15 L/min. Finally, doctor cannot but decide to improve oxygen support by Bipap ventilation during February 7–13 (Table S2) and fortunately, the patient's situation get better gradually since then. On February 17, the beside chest radiography showed improved notedly, compared to the imaging of February 3 (Figure 1E,F). On the same day, the high flow nasal cannula was adjusted to 7 L/min. It was on February 21, twenty‐eighth day from the onset of the disease, when the patient got normal laboratory index and nasal cannula was 3 L/min.

On March 2, the special IgM and lgG antibody for SARS‐CoV‐2 was successfully harvested to test the pathogeny related with the novel coronavirus by Bestnovo detection kit (Methods S1) and both results appear positive ten minutes later (Figure 2E). As for the total serum antibody concentration, there was no significant fluctuation between IgM 0.51 g/L (normal 0.4–2.3 g/L) and IgG 15.89 g/L (normal range 7‐16 g/L) on January 31 and IgM 0.87 g/L and IgG 15.34 g/L on March 9.

## 3 DISCUSSION

Looking through the disease process of the patient, even though real‐time RT‐PCR assay was taken three times to detect the SARS‐CoV‐2 from his blood, swab and faeces, separately, the results were all negative. The explanation may be that the viral load of these three samples was not enough to appear positive, because the nucleic acid detection positive rate was generally around 50%‐70% by using the pharyngeal swabs. And if the patient was then tested by using lower respiratory tract secretions which have higher positive rate, maybe the positive result occurred.

Nevertheless, through comprehensive consideration of his clinical feature and contact history with the patient of SARS‐CoV‐2 infection, the patient was highly suspected with the infection of SARS‐CoV‐2 and therapied according to the guidelines for diagnosis and treatment of SARS‐CoV‐2. During the late stage of disease, the patient was confirmed by lgM/lgG serum antibody detection and his laboratory index got normal, as well as his beside chest radiography showed improved notedly. What can be explained from this point is that the serum antibody detection is also a very important evidence to diagnosis the infection of SARS‐CoV‐2 among patients with typical clinical features. Now, this strategy has been officially used as a diagnostic basis by the Chinese Health Department.

In addition to the advantages of convenience and accuracy, lgM/lgG serum antibody detection can also liberate a large number of suspected patients and close contacts in a short time, and screen out asymptomatic infected persons. Through the combination of nucleic acid detection, we can accurately detect which people are infected, so as to take scientific measures. In this way, while improving the diagnosis rate of patients, the waste of human and material resources could be greatly reduced so as to restore economic development.

## CONFLICTS OF INTEREST

The authors declare no conflict of interest.

## AUTHOR CONTRIBUTIONS

HL and YS collected, analysed and interpreted the data. BW did the total immunoglobulin tests of lgM/lgG serum antibody. JG and JP conceived and supervised the study. HL and Y.S wrote the manuscript. JG finalized the manuscript. All authors read and approved the final manuscript.

## Supporting information

Supplementary Material

Table S1‐S2

## Data Availability

All data, models, and code generated or used during the study appear in the submitted article.
