# Supplementary material for: SARS‐CoV‐2 lgM/lgG antibody detection confirms the infection after three negative nucleic acid detection
Source: J Cell Mol Med. 2020 May 19;24(14):8262–5. doi: 10.1111/jcmm.15275 (PMC7280606; doi:10.1111/jcmm.15275)
Supplement: Supplementary file 1 — Supplementary Material [file JCMM-24-8262-s001.docx]

**Supplementary Methods**

**The total immunoglobulin tests**

The total serum immunoglobulin was tested by transmission immunoturbidimetry (Diasys, Germany). Immunoturbidimetry allows quantitative determination of proteins by specific antigen-antibody reaction. This interaction causes agglutination leading to turbidity, directly influencing the intensity of the transmitted light. The latter is measured photometrically and correlates with the concentration of the analyte in the sample.

**The preparation of specific antibody**

The targeted sequence was amplified by primers designed according to the reported 2019-nCoV sequence via polymerase chain reaction (PCR) and then cloned into the cloning vector of Escherichia coli (Pgem-7zf (+)). After verified by Sanger sequencing, the targeted sequence was cloned into expression vector of Escherichia coli (pBV220) to induce protein expression. The expressed protein in the form of inclusion body was verified via protein electrophoresis, showing the same molecular weight as that of predicted. Recombinant protein displayed reactogenicity in the Western Blot and DIFGA.

**The structure of colloidal gold strips
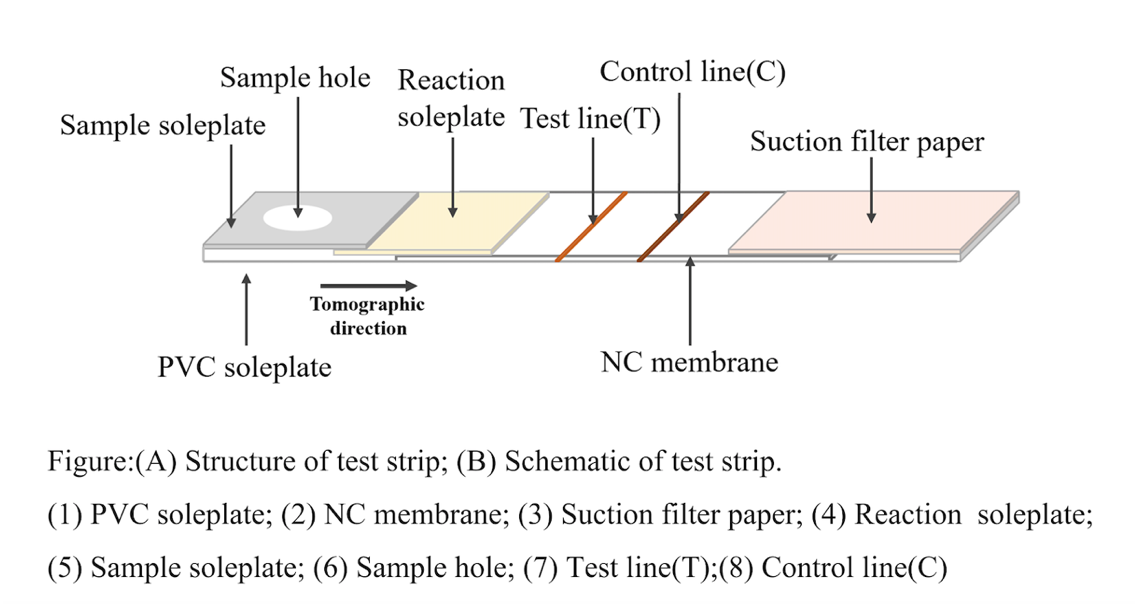
**

Fractions of protein S1 and S2 were embedded in T line

**Protein S (S1 and S2) nucleotide sequence and protein amino acid sequence from NC_045512.2**

1 ATGTTTGTTTTTCTTGTTTTATTGCCACTAGTCTCTAGTCAGTGTGTTAATCTTACAACC

1 M F V F L V L L P L V S S Q C V N L T T

61 AGAACTCAATTACCCCCTGCATACACTAATTCTTTCACACGTGGTGTTTATTACCCTGAC

21 R T Q L P P A Y T N S F T R G V Y Y P D

121 AAAGTTTTCAGATCCTCAGTTTTACATTCAACTCAGGACTTGTTCTTACCTTTCTTTTCC

41 K V F R S S V L H S T Q D L F L P F F S

181 AATGTTACTTGGTTCCATGCTATACATGTCTCTGGGACCAATGGTACTAAGAGGTTTGAT

61 N V T W F H A I H V S G T N G T K R F D

241 AACCCTGTCCTACCATTTAATGATGGTGTTTATTTTGCTTCCACTGAGAAGTCTAACATA

81 N P V L P F N D G V Y F A S T E K S N I

301 ATAAGAGGCTGGATTTTTGGTACTACTTTAGATTCGAAGACCCAGTCCCTACTTATTGTT

101 I R G W I F G T T L D S K T Q S L L I V

361 AATAACGCTACTAATGTTGTTATTAAAGTCTGTGAATTTCAATTTTGTAATGATCCATTT

121 N N A T N V V I K V C E F Q F C N D P F

421 TTGGGTGTTTATTACCACAAAAACAACAAAAGTTGGATGGAAAGTGAGTTCAGAGTTTAT

141 L G V Y Y H K N N K S W M E S E F R V Y

481 TCTAGTGCGAATAATTGCACTTTTGAATATGTCTCTCAGCCTTTTCTTATGGACCTTGAA

161 S S A N N C T F E Y V S Q P F L M D L E

541 GGAAAACAGGGTAATTTCAAAAATCTTAGGGAATTTGTGTTTAAGAATATTGATGGTTAT

181 G K Q G N F K N L R E F V F K N I D G Y

601 TTTAAAATATATTCTAAGCACACGCCTATTAATTTAGTGCGTGATCTCCCTCAGGGTTTT

201 F K I Y S K H T P I N L V R D L P Q G F

661 TCGGCTTTAGAACCATTGGTAGATTTGCCAATAGGTATTAACATCACTAGGTTTCAAACT

221 S A L E P L V D L P I G I N I T R F Q T

721 TTACTTGCTTTACATAGAAGTTATTTGACTCCTGGTGATTCTTCTTCAGGTTGGACAGCT

241 L L A L H R S Y L T P G D S S S G W T A

781 GGTGCTGCAGCTTATTATGTGGGTTATCTTCAACCTAGGACTTTTCTATTAAAATATAAT

261 G A A A Y Y V G Y L Q P R T F L L K Y N

841 GAAAATGGAACCATTACAGATGCTGTAGACTGTGCACTTGACCCTCTCTCAGAAACAAAG

281 E N G T I T D A V D C A L D P L S E T K

901 TGTACGTTGAAATCCTTCACTGTAGAAAAAGGAATCTATCAAACTTCTAACTTTAGAGTC

301 C T L K S F T V E K G I Y Q T S N F R V

961 CAACCAACAGAATCTATTGTTAGATTTCCTAATATTACAAACTTGTGCCCTTTTGGTGAA

321 Q P T E S I V R F P N I T N L C P F G E

1021 GTTTTTAACGCCACCAGATTTGCATCTGTTTATGCTTGGAACAGGAAGAGAATCAGCAAC

341 V F N A T R F A S V Y A W N R K R I S N

1081 TGTGTTGCTGATTATTCTGTCCTATATAATTCCGCATCATTTTCCACTTTTAAGTGTTAT

361 C V A D Y S V L Y N S A S F S T F K C Y

1141 GGAGTGTCTCCTACTAAATTAAATGATCTCTGCTTTACTAATGTCTATGCAGATTCATTT

381 G V S P T K L N D L C F T N V Y A D S F

1201 GTAATTAGAGGTGATGAAGTCAGACAAATCGCTCCAGGGCAAACTGGAAAGATTGCTGAT

401 V I R G D E V R Q I A P G Q T G K I A D

1261 TATAATTATAAATTACCAGATGATTTTACAGGCTGCGTTATAGCTTGGAATTCTAACAAT

421 Y N Y K L P D D F T G C V I A W N S N N

1321 CTTGATTCTAAGGTTGGTGGTAATTATAATTACCTGTATAGATTGTTTAGGAAGTCTAAT

441 L D S K V G G N Y N Y L Y R L F R K S N

1381 CTCAAACCTTTTGAGAGAGATATTTCAACTGAAATCTATCAGGCCGGTAGCACACCTTGT

461 L K P F E R D I S T E I Y Q A G S T P C

1441 AATGGTGTTGAAGGTTTTAATTGTTACTTTCCTTTACAATCATATGGTTTCCAACCCACT

481 N G V E G F N C Y F P L Q S Y G F Q P T

1501 AATGGTGTTGGTTACCAACCATACAGAGTAGTAGTACTTTCTTTTGAACTTCTACATGCA

501 N G V G Y Q P Y R V V V L S F E L L H A

1561 CCAGCAACTGTTTGTGGACCTAAAAAGTCTACTAATTTGGTTAAAAACAAATGTGTCAAT

521 P A T V C G P K K S T N L V K N K C V N

1621 TTCAACTTCAATGGTTTAACAGGCACAGGTGTTCTTACTGAGTCTAACAAAAAGTTTCTG

541 F N F N G L T G T G V L T E S N K K F L

1681 CCTTTCCAACAATTTGGCAGAGACATTGCTGACACTACTGATGCTGTCCGTGATCCACAG

561 P F Q Q F G R D I A D T T D A V R D P Q

1741 ACACTTGAGATTCTTGACATTACACCATGTTCTTTTGGTGGTGTCAGTGTTATAACACCA

581 T L E I L D I T P C S F G G V S V I T P

1801 GGAACAAATACTTCTAACCAGGTTGCTGTTCTTTATCAGGATGTTAACTGCACAGAAGTC

601 G T N T S N Q V A V L Y Q D V N C T E V

1861 CCTGTTGCTATTCATGCAGATCAACTTACTCCTACTTGGCGTGTTTATTCTACAGGTTCT

621 P V A I H A D Q L T P T W R V Y S T G S

1921 AATGTTTTTCAAACACGTGCAGGCTGTTTAATAGGGGCTGAACATGTCAACAACTCATAT

641 N V F Q T R A G C L I G A E H V N N S Y

1981 GAGTGTGACATACCCATTGGTGCAGGTATATGCGCTAGTTATCAGACTCAGACTAATTCT

661 E C D I P I G A G I C A S Y Q T Q T N S

2041 CCTCGGCGGGCACGTAGTGTAGCTAGTCAATCCATCATTGCCTACACTATGTCACTTGGT

681 P R R A R S V A S Q S I I A Y T M S L G

**S1/S2**

2101 GCAGAAAATTCAGTTGCTTACTCTAATAACTCTATTGCCATACCCACAAATTTTACTATT

701 A E N S V A Y S N N S I A I P T N F T I

2161 AGTGTTACCACAGAAATTCTACCAGTGTCTATGACCAAGACATCAGTAGATTGTACAATG

721 S V T T E I L P V S M T K T S V D C T M

2221 TACATTTGTGGTGATTCAACTGAATGCAGCAATCTTTTGTTGCAATATGGCAGTTTTTGT

741 Y I C G D S T E C S N L L L Q Y G S F C

2281 ACACAATTAAACCGTGCTTTAACTGGAATAGCTGTTGAACAAGACAAAAACACCCAAGAA

761 T Q L N R A L T G I A V E Q D K N T Q E

2341 GTTTTTGCACAAGTCAAACAAATTTACAAAACACCACCAATTAAAGATTTTGGTGGTTTT

781 V F A Q V K Q I Y K T P P I K D F G G F

2401 AATTTTTCACAAATATTACCAGATCCATCAAAACCAAGCAAGAGGTCATTTATTGAAGAT

801 N F S Q I L P D P S K P S K R S F I E D

2461 CTACTTTTCAACAAAGTGACACTTGCAGATGCTGGCTTCATCAAACAATATGGTGATTGC

821 L L F N K V T L A D A G F I K Q Y G D C

2521 CTTGGTGATATTGCTGCTAGAGACCTCATTTGTGCACAAAAGTTTAACGGCCTTACTGTT

841 L G D I A A R D L I C A Q K F N G L T V

2581 TTGCCACCTTTGCTCACAGATGAAATGATTGCTCAATACACTTCTGCACTGTTAGCGGGT

861 L P P L L T D E M I A Q Y T S A L L A G

2641 ACAATCACTTCTGGTTGGACCTTTGGTGCAGGTGCTGCATTACAAATACCATTTGCTATG

881 T I T S G W T F G A G A A L Q I P F A M

2701 CAAATGGCTTATAGGTTTAATGGTATTGGAGTTACACAGAATGTTCTCTATGAGAACCAA

901 Q M A Y R F N G I G V T Q N V L Y E N Q

2761 AAATTGATTGCCAACCAATTTAATAGTGCTATTGGCAAAATTCAAGACTCACTTTCTTCC

921 K L I A N Q F N S A I G K I Q D S L S S

2821 ACAGCAAGTGCACTTGGAAAACTTCAAGATGTGGTCAACCAAAATGCACAAGCTTTAAAC

941 T A S A L G K L Q D V V N Q N A Q A L N

2881 ACGCTTGTTAAACAACTTAGCTCCAATTTTGGTGCAATTTCAAGTGTTTTAAATGATATC

961 T L V K Q L S S N F G A I S S V L N D I

2941 CTTTCACGTCTTGACAAAGTTGAGGCTGAAGTGCAAATTGATAGGTTGATCACAGGCAGA

981 L S R L D K V E A E V Q I D R L I T G R

3001 CTTCAAAGTTTGCAGACATATGTGACTCAACAATTAATTAGAGCTGCAGAAATCAGAGCT

1001 L Q S L Q T Y V T Q Q L I R A A E I R A

3061 TCTGCTAATCTTGCTGCTACTAAAATGTCAGAGTGTGTACTTGGACAATCAAAAAGAGTT

1021 S A N L A A T K M S E C V L G Q S K R V

3121 GATTTTTGTGGAAAGGGCTATCATCTTATGTCCTTCCCTCAGTCAGCACCTCATGGTGTA

1041 D F C G K G Y H L M S F P Q S A P H G V

3181 GTCTTCTTGCATGTGACTTATGTCCCTGCACAAGAAAAGAACTTCACAACTGCTCCTGCC

1061 V F L H V T Y V P A Q E K N F T T A P A

3241 ATTTGTCATGATGGAAAAGCACACTTTCCTCGTGAAGGTGTCTTTGTTTCAAATGGCACA

1081 I C H D G K A H F P R E G V F V S N G T

3301 CACTGGTTTGTAACACAAAGGAATTTTTATGAACCACAAATCATTACTACAGACAACACA

1101 H W F V T Q R N F Y E P Q I I T T D N T

3361 TTTGTGTCTGGTAACTGTGATGTTGTAATAGGAATTGTCAACAACACAGTTTATGATCCT

1121 F V S G N C D V V I G I V N N T V Y D P

3421 TTGCAACCTGAATTAGACTCATTCAAGGAGGAGTTAGATAAATATTTTAAGAATCATACA

1141 L Q P E L D S F K E E L D K Y F K N H T

3481 TCACCAGATGTTGATTTAGGTGACATCTCTGGCATTAATGCTTCAGTTGTAAACATTCAA

1161 S P D V D L G D I S G I N A S V V N I Q

3541 AAAGAAATTGACCGCCTCAATGAGGTTGCCAAGAATTTAAATGAATCTCTCATCGATCTC

1181 K E I D R L N E V A K N L N E S L I D L

3601 CAAGAACTTGGAAAGTATGAGCAGTATATAAAATGGCCATGGTACATTTGGCTAGGTTTT

1201 Q E L G K Y E Q Y I K W P W Y I W L G F

3661 ATAGCTGGCTTGATTGCCATAGTAATGGTGACAATTATGCTTTGCTGTATGACCAGTTGC

1221 I A G L I A I V M V T I M L C C M T S C

3721 TGTAGTTGTCTCAAGGGCTGTTGTTCTTGTGGATCCTGCTGCAAATTTGATGAAGACGAC

1241 C S C L K G C C S C G S C C K F D E D D

3781 TCTGAGCCAGTGCTCAAAGGAGTCAAATTACATTACACATAA

1261 S E P V L K G V K L H Y T *
