# Supplementary material for: SARS‐CoV‐2 lgM/lgG antibody detection confirms the infection after three negative nucleic acid detection
Source: J Cell Mol Med. 2020 May 19;24(14):8262–5. doi: 10.1111/jcmm.15275 (PMC7280606; doi:10.1111/jcmm.15275)
Supplement: Supplementary file 2 — Table S1‐S2 [file JCMM-24-8262-s002.docx]

**Supplementary Tables**

Table 1. Body temperature and laboratory data for the patient.

|  | **Body Temperature** | **Leukocyte** | **Neutrophils** | **Lymphocytes** | **CRP** | **ESR** | **PaO2** |
| --- | --- | --- | --- | --- | --- | --- | --- |
| **Jan 26** | 38.7°C | 5.75*10^9/L | 75.7% | 13.6% | / | / | / |
| **Jan 28** | 39℃ | 4.99*10^9/L | 67.8% | 19% | / | / | / |
| **Jan 31** | 37.4 | 7.13*10^9/L | 75.7% | 10.9% | 68.8  mmol/L | 58  mm/H | 76 mmHg  (nasal cannula 15L/min) |
| **Feb 1** | 36.6 | 12.20*10^9/L | 91.7% | 3.0% | 137  mmol/L | 62  mm/H | / |
| **Feb 2** | 38.0 | 16.84*10^9/L | 89.7% | 4.5% | 164.9  mmol/L | 63  mm/H | 77 mmHg  (nasal cannula 15L/min) |
| **Feb 3** | 37.3 | 15.73*10^9/L | 86.6% | 5.8% | 156.2  mmol/L | 58  mm/H | 98 mmHg  (nasal cannula 15L/min) |
| **Feb 4** | 37.3 | 10.95*10^9/L | 80.3% | 8.0% | 113.7  mmol/L | 76  mm/H | 76 mmHg  (nasal cannula 15L/min) |
| **Feb 5** | 37.3 | 9.9*10^9/L | 73.4% | 12.4% | 69.9  mmol/L | 76  mm/H | 82 mmHg  (nasal cannula 15L/min) |
| **Feb 7** | 37.3 | 16.84*10^9/L | 89.7% | 4.5% | 164.9  mmol/L | 63  mm/H | 77 mmHg  (nasal cannula 15L/min) |
| **Feb 8** | 36.9 | 12.99*10^9/L | 76.6% | 12.4% | 61.6  mmol/L | 60  mm/H | 120 mmHg  (Bipap 8L/min) |
| **Feb 9** | 36.8 | 9.42*10^9/L | 74.8% | 15.3% | 37.2  mmol/L | 73  mm/H | 103 mmHg  (Bipap 7L/min) |
| **Feb 10** | 36.8 | 7.22*10^9/L | 68.5% | 20.6% | 21.3  mmol/L | 44  mm/H | 135 mmHg  (Bipap 7L/min) |
| **Feb 11** | 37.3 | 7.87*10^9/L | 68.3% | 21.5% | 13.3  mmol/L | 54  mm/H | 99 mmHg  (Bipap 6L/min) |
| **Feb 13** | 36.9 | 6.35*10^9/L | 43.4% | 24.3% | 5.9  mmol/L | 21  mm/H | 107 mmHg  (nasal cannula 8 L/min) |
| **Feb 15** | 36.9 | 6.40*10^9/L | 61.9% | 24.7% | 3.8  mmol/L | 40  mm/H | 102 mmHg  (nasal cannula 15L/min) |
| **Feb 17** | 37.1 | 6.12*10^9/L | 60.9% | 23.5% | 2.5  mmol/L | 40  mm/H | 89 mmHg  (nasal cannula 7L/min) |
| **Feb 19** | 36.9 | 6.70*10^9/L | 61.9% | 22.4% | 2.4  mmol/L | 37  mm/H | 134 mmHg  (nasal cannula 6L/min) |
| **Feb 21** | 36.8 | 6.25*10^9/L | 61.3% | 23% | 2.0  mmol/L | 46  mm/H | 95 mmHg  (nasal cannula 3 L/min) |

Table 2. Clinical therapy for the patient.

| **Treatment** | | | | | | | | | |
| --- | --- | --- | --- | --- | --- | --- | --- | --- | --- |
| **Start Time** | **Levofloxacin** | **oseltamivir** | **methylprednisolone** | **immunoglobulin** | **doxycycline** | **piperacillin tazobactam** | **ertapenem** | **nasal cannula** | **Bipap ventilation** |
| **Jan 29** | 0.5g po qd | 75mg po bid  (to Feb 11) | 40mg po qd  (to Feb 1) | 10g iv qd  (to Feb 3) |  |  |  | 2 L/min |  |
| **Jan 30** | 0.5g iv qd  (to Feb 19) |  |  |  |  |  |  | 15 L/min  (to Feb 6) |  |
| **Jan 31** |  |  |  |  |  |  |  |  |  |
| **Feb 2** |  |  | 30mg po qd |  | 0.1g po q12h  (to Feb 10) |  |  |  |  |
| **Feb 3** |  |  | 20mg po qd |  |  | 4.5g iv q8h  (to Feb 5) |  |  |  |
| **Feb 6** |  |  | 40mg po qd  (to Feb 8) |  |  |  | 1g iv qd  (to Feb 7) |  |  |
| **Feb 7** |  |  |  |  |  |  |  |  | ST 8-15T/min  15 cmH_2_O IPAP & 5cm cmH_2_O EPAP  (to Feb 13) |
| **Feb 13** |  |  |  |  |  |  |  | 5-8 L/min |  |
| **Feb 21** |  |  |  |  |  |  |  | 3 L/min |  |
